# The emergence of the postgenomic gene

**DOI:** 10.1007/s13194-022-00446-0

**Published:** 2022-02-15

**Authors:** Francesca Bellazzi

**Affiliations:** Department of Philosophy, University of Bristol, Bristol, UK

**Keywords:** Postgenomic genes, Emergence, Robustness

## Abstract

The identity and the existence of genes has been challenged by postgenomic discoveries. Specifically, the consideration of molecular and cellular phenomena in which genes are embedded has proved relevant for their understanding. In response to these challenges, I will argue that the complexity of genetic phenomena supports the weak emergence of genes from the DNA. In Section 2, I will expose what genes are taken to be in the postgenomic world. In Section 3, I will present the relevant account of emergence. I consider weak emergence as in Franklin and Knox (*Studies for the History and Philosophy of Modern Physics, 64*, 68–78, 2018), for which a phenomenon is emergent when it displays novelty and robustness. In Section 4, I will argue that genes are weakly emergent since they are novel, improving explanations, and robust in respect to some perturbations. Then, I will conclude in Section 5 that genes’ emergence is a way to allow genes’ flexibility and context dependency, without compromising their existence.

## Introduction

Genes are ways in which cells utilise available template resources to create the biomolecules that are needed in a specific place at a specific time: genes are things an organism can do with its genome. (Griffiths & Stotz, 2013, 75).

Do genes really exist? With the discoveries that followed the genome project, this question has proved difficult to answer due to the complexity of genetic phenomena. The various processes involving nucleic acids have shown the limits of characterizing genes only in terms of a determinate physical stretch of DNA and a specific molecular product (also Fogle, 2010; Hall, 2001). This has resulted in a sceptical or deflationary approach to genes, which can be considered mere useful tools for genomic analysis. Nevertheless, it is hard to accept such a view, as genes still retain a central role in many subdisciplines of contemporary biology. In this paper, I will argue in favour of a positive answer: genes exist, but they emerge during the transcription processes. The result elaborates on the post-genomic awareness that genes have a proper identity only within the broader context of general molecular interactions and cellular processes (Burian, 2004; El-Hani, 2007; Fogle, 2010; Griffiths & Stotz, 2013; Hall, 2001; Scherrer & Jost, 2007). In order to understand what it takes for something to be a gene and then assess its existence, we need to stop focusing just on its molecular components, and we should consider the interactions that concern gene expression (El-Hani, 2007; Fogle, 2010; Griffiths & Stotz, 2013). The gene has its proper home in the cell and cannot be understood without it. This will be the starting point for the present analysis.

My argument in favour of the existence of genes as emergent entities will proceed in two steps. First, I will defend and clarify a definition of postgenomic genes presented in the literature by Gerstain et al. (2007) and Griffiths and Stotz (2013). According to this proposal, genes are not mere linear sequences of DNA, but complex entities that depend on a context of inter-, intra-, and extra- cellular factors.^1^ Then, I will argue that genes so-characterised are weakly emergent from the DNA basis, insofar as they display explanatory novelty and robustness. This account of emergence will be ontological and motivated on moderately naturalistic grounds.^2^

In detail, this novel approach has different implications. First, it applies the metaphysics of science and the concept of emergence to a complicated issue in philosophy of biology and might help disentangle some of the tensions within the gene debate. This approach has been producing fruitful results in relation to other sciences, and it can be the same for the philosophy of biology. Furthermore, biologists who have been experiencing the problems of the gene concept might benefit from this account. It will provide them with a novel framework that allows for both gene identity and genes’ contextual dependence. This can provide a better understanding of the phenomena under discussion. Specifically, the consideration of the context in which genes exist can be relevant when theorising on genetic phenomena. Also, this allows scientists with a realist tendency towards their discipline to accept the existence of genes within the manifested complexity. Second, this case study can be of interest to all the philosophers working in the field of emergence in the sciences. I apply here a novel account of weak emergence mostly proposed in the philosophy of physics, as in Franklin and Knox (2018), to cases in biology. This view is a significant proposal of weak emergence because it is sensitive to scientific practice, being easily applied to concrete examples from science, and compatible with non-eliminativist ontological reductionism. This approach will allow us to retain the gene as being materially constituted by nucleic acids, while maintaining a type difference between the gene and its underlying basis.

In Section 2, I will present the gene in the context of the post-genomic era. In the 1960s, the gene was defined as a precise and ordered sequence of nucleotides that encodes the primary structure of a polypeptide or a functional RNA molecule. Nevertheless, discoveries of the last four decades made the identification of a 1:1 correspondence between genes and stretches of DNA impossible. The postgenomic gene turns out to be highly dependent on the molecular and cellular context. The complexities involved in the current status of the postgenomic genes can be taken into account because, I will argue, genes weakly emerge from the underlying nucleic acid basis.

In Section 3, I will introduce the concept of ontological *weak emergence* relevant to the case in question, presented by Franklin and Knox (2018). According to this account, a phenomenon is claimed to be *weakly emergent* when it has properties that are (i) explanatorily novel, as they improve explanations; (ii) robust relative to some lower-level perturbations, here interpreted in relation to multiple realisability and multiple constitution.^3^ This type of emergence is ontological and incompatible with eliminativism, but nevertheless compatible with ontological reductionism: the *token* of the emergent phenomenon will be identical with *some* lower-level phenomena at the time considered. This is coherent with the strict relation between DNA and genes acknowledged by scientific practice. Furthermore, the focus on robustness, a well-acknowledged feature of genetic phenomena, makes it particularly suitable for its application in the life sciences (see Boone, 2018; Eronen, 2015).

In Section 4, I will show that genes are *weakly emergent* in the aforementioned sense. Even if there is substantial conceptual unclarity about the definition of genes two crucial features are identifiable. Broadly, a gene is a part of DNA that has the property of being transcribed into specific molecular products (Griffiths & Stotz, 2013). This entails that there are two crucial properties of the gene, one material and one functional. First, it has to be composed of nucleic acids (DNA or RNA),^4^ second it has to be actively transcribed. This allows us to identify a material component of the gene: the sequence strictly involved in transcription, and, according to most models, this includes also the promoter region (or TATA box) (Fogle, 2010, 6; Griffiths & Stotz, 2013, 71).^5^ It also permits us to identify a functional component: the gene has the function of being transcribable. This is what discriminates genes from other regions of DNA. Furthermore, this second property is not an intrinsic property of a *linear* contiguous sequence of DNA, but it depends on a full set of molecular and cellular interactions. Here, I will propose a way in which the gene retains its identity despite this context dependence. I will first address some of the phenomena that made the identification of genes with linear stretches of DNA molecules more complicated. Then, I will argue why genes satisfy novelty and robustness. To state it more clearly, a gene emerges from a precise stretch of DNA when it is expressed, thanks to the interactions that make it novel and robust. I will conclude in Section 5 that the emergence of the postgenomic gene allows us to retain the gene as an existent phenomenon, embracing its flexible and context-dependent identity.

## The postgenomic gene

The 1960/70s were the golden years of molecular genetics and it seemed clear that genes were nothing more than segments of DNA located on a chromosome that give rise to a particular amino acid sequence. This correspondence was formulated as the *Crick-sequence hypothesis*: each codon, sequence of three bases, specifies only one amino acid and a gene is a sequence of codons that specifies for a polypeptide (Griffiths & Stotz, 2013). With subsequent discoveries of the 1980s, genes were understood as “open reading frameworks” (ORFs): DNA frameworks open to be read (Gerstain et al., 2007). This facilitated research at the time, as the gene was identified as a well-defined and structured stretch of DNA, with clear borders and a singular function. However, things turned out to be more complicated. The production of new technologies to sequence genomes and the advances in molecular biology of the last 40 years have disclosed peculiar genetic phenomena (El-Hani, 2007; Fox Keller, 2000; Griffiths & Stotz, 2013; Hall, 2001; Meyer et al., 2013). Specifically, the sequence of entire genomes and the study of eukaryotic ones revealed that the Crick-sequence hypothesis was simplistic and further developments of genetics have made it impossible for genes to be a merely contiguous DNA segment co-linear with the product derived (Fogle, 2010; Perini, 2011; Rheinberger et al., 2015). These developments compromised the material identity of the gene as a discrete stretch of DNA and showed the inefficiency of its identification in mere material terms (Falk, 2010). With the new century, the gene is now a concept in tension. In 2007, El-Hani spoke of the crisis of the gene that finds itself between “the cross and the sword” because of a series of complex phenomena, such as split genes, alternative splicing, overlapping and nested genes (Griffiths & Stotz, 2013).

These phenomena illustrate how the gene is embedded in a series of complex and different interactions that surround it. And despite the temptation to consider the gene a dead road, there is a consensus on the need of relocating it within its cellular and organismal context (as Beurton, 2010 argues). It seems true that “the gene is not dead, but alive and well, even though orphaned, homeless, and seeking a haven from which to steer a course to its natural home, *the cell* as a fundamental morphogenetic unit” (Hall, 2001, 225–228; emph. added). Given the number of publications, experiments and biological scientific practice that even today involves gene-talk, it seems mandatory to understand the gene keeping together its molecular aspect and its context dependency (as Burian, 2004 argues).^6^ As a reaction, many have been re-thinking and re-defining the gene concept in a variety of ways,^7^ trying to accommodate theoretical and practical requirements. Generalising, we can indicate two main ways to re-think the gene concept. On the one hand, we find what has been called “the nominal gene” (Burian, 2004; Griffiths & Stotz, 2006, 2013). This is a “relatively conservative conception of gene” as stretch of DNA with precise nucleotides sequences that encode a specific product (Griffiths & Stotz, 2013, 66). As suggested by its nominal component, such view of genes is highly operational and has a deflationary connotation. Related to this, we find the so-called “consensus gene” as any “general pattern of biochemical architecture and process” that shares the features of exemplary gene cases according to scientific practice and empirical evidence (Fogle, 2010). On the other hand, we find a more realist understanding of the “postgenomic genes” as “images of the target produced molecules” (Griffiths & Stotz, 2013, 75). Such genes can only be fully understood in their environment and in their context of action (Falk, 2010; Griffiths & Stotz, 2006). Here, I will start from this second approach to the genes. Such a conception allows to retain a correspondence between gene and products, but nevertheless it does not imply the fixity of identifying the gene with a given DNA sequence.

### What is a postgenomic gene?

Let us now look more closely at postgenomic genes. In contemporary biological textbooks, genes are broadly defined by their function and by their composition. For instance, “*The gene is a unit of information and corresponds to a discrete segment of DNA that encodes the amino acid sequence of a polypeptide*” (Fletcher & Hickey, 2012).^8^ However, such a definition provides just a starting point to understand the gene in detail, and it is close to the aforementioned nominal gene concept. Let us now look at a more advanced definition, proposed in the context of the ENCODE analysis: “*A gene is a union of genomic sequences encoding a coherent set of potentially overlapping functional products*” (Gerstain et al., 2007). Something is defined as gene if it is composed of *a* genomic sequence and it is transcribed into a coherent set of transcripts. Nevertheless, the gene is not a linear sequence, but a union of different genomic sequences, and transcription cannot be understood without the complex molecular and cellular context that allows for it. Trying to account for such a complexity, Griffiths and Stotz (2013) elaborate the proposal of Gerstain et al. (2007) and define postgenomic genes as “images of the target molecule” that can be produced only in a wider system of interactions and environment. This makes the genes not simple contiguous stretches of DNA, but rather sets of DNA sequences with a specific function that are not necessarily contiguous; as far as their definition is concerned, “function and structure are inseparable” (Fogle, 2010, 5). The structural or material component of the gene normally includes not only the finally transcribed sequence, but often also the promoter (or TATA box) is accepted as a consensus feature of a gene (Fogle, 2010, 6). In addition to it there might be other regions involved that are essential for the activation of the gene and the regulation of its transcription (Griffiths & Stotz, 2006, 2013).

Here, two questions might rise. The first is which parts of the DNA should be considered as genes. I claim that such an answer can only come from empirical scientific practice. The empirical evidence concerning which parts of the DNA regions are counted as genes is constantly changing and improving, and the answer to this question has to be provided by science.^9^ For instance, it is actual scientific practice that can tell when some regulatory parts of the DNA can be included in the different genes. This might also be dependent on individual cases. The second question asks what it takes for something to be considered a gene at all and whether such entities exist. This is what I will consider here.

For the purposes of the account, we are interested in the identification of a definition that points toward what makes a phenomenon different from something else. Combining the aforementioned definitions, we notice that the gene has a two-fold one. The gene has a structural or material component as a region of DNA. Conversely, not every region of DNA is a gene. Only those regions actively involved in transcription as “images” of the target molecules are genes. And a gene is what has the property of being transcribed into a RNA, which can then encode the primary structure of a polypeptide or can play a regulatory, structural or catalytic function. Thus, there are two crucial properties of the gene: i) it has to be composed of DNA; ii) it has to be transcribed or to be strictly involved in transcription (Fogle, 2010; Griffiths & Stotz, 2013). Transcription, however, is not a self-subsistent phenomenon, but it is rather a reactive one: it happens only within the right circumstances and thanks to a set of interactions that operate at different levels (Griffiths & Stotz, 2006, 2013). In functional terms, a gene is a part of DNA that *has the function* of being transcribed or being involved in transcription (according to the accounts considered). This implies that the gene is a *context-dependent phenomenon*: “a function is always a role in something and a contribution to something” (Germain et al., 2014) and it acquires its proper identity only when considered within the cellular context in which exists. In the case of genes, the transcriptional aspect is what makes them an “image” of the molecular product. What makes some parts of the DNA *a gene* is its involvement in transcription. A further support to the relevance of the functional component comes from the fact that genes are normally divided into two functional subtypes. The first are genes that encode the RNA for the amino acid sequence of a polypeptide; the second are genes that encode an RNA that regulates a variety of different cellular processes or play other functional roles (as in Fletcher & Hickey, 2012; Meyer et al., 2013). Accepting that genes are functionally defined *does not imply* that they are *exclusively functional*. But here I claim that a minimum requirement for something to be a gene is to have a functional property and *some* underlying material DNA basis.^10^ The importance of the functional aspect brings with it considerations about the context and conditions in which such a function is realised. Without molecular interactions, the DNA is an inert molecule that cannot be transcribed. The gene can only be properly understood taking into account the system that surrounds it. This implies that the core property of genes is relational: it exists only when specific interactions between the DNA and the context happen.

Such complexity and context dependency might lead towards a more deflationary view of the genes, however the present paper wants to argue in the opposite direction. It aims at providing a novel framework that allows us to accept the existence of genes as entities with a material component (for instance, DNA) and a context-dependent functional one. I will do so by arguing that genes are more complex than their DNA bases and, in having a functional component, they *are weakly emergent.*^11^ This approach will be of interest to scientists with a more realist attitude towards their discipline as it will provide them with an account in favour of genes existence. Moreover, such a view can provide more clarity when theorising genetic phenomena: genes exist as emergent entities in specific contexts.

## Ontological emergence as novelty and robustness

In which sense then are the genes emergent? The debate on emergence is wide in approaches and topics,^12^ and for the purposes of this paper, I will focus only on an account recently proposed by Franklin and Knox (2018). This has been advanced as a form of ontological weak emergence, and it elaborates on previous works of Knox (2016) and Butterfield (2011). I find it to be among the best recent formulations of weak emergence, as it is sensitive to scientific practice and compatible with non-eliminativist ontological reductionism.

Emergence can be generally defined as a combination of autonomy and dependency: the emergent phenomenon has to be autonomous in some sense, but nevertheless dependent on its basis. The formulation of Franklin and Knox (2018) captures well these two aspects. The autonomous component of emergence is given by the features of novelty and robustness. Nevertheless, such an account is compatible with forms of ontological non-reductivism, which allows for the dependency component.^13^ This is because higher-level entities exist (so they are *not eliminable*), but they are reducible to the lower-level entities that realise them^14^: the emergent entities will be identical at the time considered with *some* of the lower-level features.^15^ Moreover, the combination of epistemic and ontological criteria, novelty and robustness, makes it coherent and sensitive to scientific practice and scientific discoveries. This is because it is easily applied to concrete case studies. Specifically, it can be applied to instances in which scientific practice takes the lower-level to be token identical with the emergent entities, even if there is a difference in type, as it can happen in genetics. Furthermore, it acknowledges the relevance of robustness, a feature of biological systems that is playing a crucial role in the discussion in the biological sciences (see Boone, 2018; Eronen, 2015). This contemporary proposal of weak emergence has been presented as the only one able to capture a complicated phenomenon, the emergence of phonons, and the authors claim that this account may be extended and applied to “many other instances of emergence across science” (Franklin & Knox, 2018, 68). Even if phonons and genes are different in many aspects, they both share a complexity that calls for further analysis and I will show that this account applies to the case of the gene, supporting its emergence.

Some clarifications are needed before entering into the details. Here, I will assume a simple two category ontology of properties and entities that bear the properties,^16^ and claim that something is emergent when its *definitional properties* are emergent (as in Wilson, 2015, 4). So, taken a given phenomenon (in this case, the gene), this phenomenon will be considered emergent when its definitional properties satisfy the given criteria for emergence. Moreover, when considering the relations between levels, I will assume that higher-level properties are *realised* by lower-level features, while higher-level entities are *constituted* by lower-level entities (see Gillett, 2013; Kistler, 2018).^17^ Let us now move to emergence. Weak emergence refers to the existence of higher-level features that are “realised by the lower-level ones” in a genuine way, even if every token of the property of the emergent feature is identical with *some*lower-level feature at the time considered (Wilson, 2015). Furthermore, Franklin and Knox (2018) specify that a weakly emergent phenomenon has to also display properties whose higher-level postulation improves our explanatory power, and these properties are robust. In detail, the defining properties of the phenomenon under consideration are considered emergent when they are characterised by two features^18^:

- Novelty. This feature implies that it is possible to identify the emergent property in a distinctive way from the properties held by the lower-level entities and the consideration of such a property improves explanations, leading to new ones. Accordingly, a phenomenon is emergent when it has a property whose postulation and usage in scientific theories leads to novel explanations^19^(see also Knox, 2016).
- Robustness. This feature implies that the emergent property displays stability within a certain range of perturbations, and relatively to some lower-level properties (Butterfield, 2011; Franklin & Knox, 2018). Even if the system undergoes modifications, the property nevertheless realises. Accordingly, a higher-level phenomenon is said to be robust when it displays a property that is stable within a certain range of perturbations, here interpreted in terms of multiple realisation and multiple constitution.

Let me consider the two relevant features in more detail. Novelty has a crucial epistemic component, as it is what permits *novel explanations *(Knox, 2016): the postulation of the property allows for new and better explanations compared to the postulation of only the basis that realises the property. The same can be said for the emergent phenomenon: it is novel when its postulation improves explanations, compared to postulating only the components. Even if novelty is epistemic, assuming a form of realism in science, it has further value as the explanatory novelty of properties is a good hint about the real existence of such a property in the world. This is in line with a form of moderate naturalism, for which ontological considerations should be made in dialogue with the scientific postulation of entities.^20^ The best way to account for the explanatory power of a phenomenon in a scientific theory is to postulate its ontological existence. Nevertheless, novelty is not enough on its own and it needs robustness.

Robustness is then the ontological feature of this account, in the sense that it concerns the existence of the property rather than its role in our scientific theories. And robustness implies that the property is present even under perturbations and changes in the environment. It is legitimate to ask which kind of perturbation is the one relevant for robustness. Differently from the original proposal,^21^ I will refer with “stability under perturbations” to cases in which the emergent features present *multiple realisability* and *multiple constitution*, as this reflects the meaning of robustness across the biological sciences (as in Boone, 2018).^22^ This is a relevant aspect of the proposal, as the robustness of the phenomenon can be read as a *genuine discriminator* between what is merely postulated by a theory and what the theory effectively captures of the world (see Weisberg, 2013, 156–170; Eronen, 2015). In the debate of emergentism, robustness might distinguish mere epistemic emergence from ontological emergence, that is the difference from the irreducibility of a theoretical phenomenon in a theory from its real existence in the world. Moreover, in the debate on the philosophy of genetics, it acknowledges an important feature of genetic phenomena that have always been characterised as robust (Falk, 2010). I will come back to elucidate the specific features of robustness in **4.1.4**.

## From complexity to emergence

Let us now consider whether genes are weakly emergent from their DNA basis according to the presented account. I will first present some of the phenomena that make the genes more complex entities than what was thought in the 1970s. Then, I will present why genes satisfy the two requirements for weak emergence, novelty and robustness.

As aforementioned, the main *properties* of a gene are i) the property of being constituted of some nucleic acids and ii) the property of being transcribable or actively involved in transcription (from now on **Ft**). Specifically, **Ft** has a special role as it allows for the gene’s being an “image” of the target molecule (as Griffiths & Stotz, 2013 points out)^23^ and is the *defining property* of the gene, relevant in identification and explanation in scientific practice. At first, **Ft** property had been ascribed to well individuated stretches of DNA, with barriers and, consequently, the gene had a precise structural and material identity. However, a series of complex phenomena challenged the identification of the gene with a precise continuous stretch on the DNA. In particular, there are three broad classes of phenomena that compromised this characterisation (El-Hani, 2007; Meyer et al., 2013):

- There are **one-to-many** correspondences between DNA segments and RNAs/ polypeptides. This means that the same stretch of DNA can give rise to different molecular products via complex mechanisms. Given the functional definition of the gene, this can be interpreted as the possibility of the same stretch of DNA to constitute different genes with different functions.^24^ For instance, the discovered discontinuous structure of genes can allow one gene to be contained inside another one’s intron (Gerstain et al., 2007). DNA seems then to have *multiple determinations*, where multiple determinability is the possibility of one microstructure, one stretch of DNA, to realise multiple biological properties and, in this case, to compose multiple genes (Tahko, 2020).

- There are **many-to-one** correspondences between DNA segments and RNAs/ polypeptides. This means that several different DNA segments or sequences can realise the same functional product. There are different stretches of DNA that can be “images” of the same transcribed product. Furthermore, there are cases in which small modifications in the underlying gene’s sequence do not change the transcribed product and the realisation of **Ft**. An instance of this phenomenon are synonymous mutations, changes in the DNA sequence that codes for a specific amino acid without affecting the final product, the encoded amino acid. **Ft** results then *multiply realisable*, where multiple realisability is the capacity of one property to be realised by a variety of microstructures or mechanisms. In parallel, it can be said that the gene is *multiply constituted*, as the same gene can be constituted by different stretches of DNA realising the appropriate **Ft**.

- And, at last, there can be a **lack of straight** correspondence between DNA segments and RNAs/ polypeptides. This means that there are functional products that do not arise from any specific and straightforward DNA sequence. An instance of the phenomenon is mRNA editing. In mRNA editing, the messenger RNA molecules are modified by enzymes after their synthesis on specific nucleosides. In this case, **Ft** is realised by whatever part of the DNA encodes the then modified mRNA. This might make the “image” within the postgenomic gene more distant from the final molecule, but it is nevertheless present. An instance of modifications of the mRNA is *trans-splicing*, in which the final mRNA is obtained from “two or more independently transcribed pre-mRNAs” (Griffiths & Stotz, 2013). In some of these cases, we can even notice a **many-to-many** relation in which the same sequences of DNA can then be modified and transcribed in different ways, fusing or “scrambling” the exons (ibid). These phenomena happen thanks to the complex molecular interactions that involve the gene and that makes it context-dependent.

A reaction to these phenomena can be a deflationary or merely nominal account of the genes, as they do not anymore satisfy the 1:1 relation that is supposed by classic molecular genetics and their clear identification is difficult.^25^ However, an alternative answer can be provided if we embrace the context dependency of the genes and we consider its definitional property **Ft** as relational. Genes are genes because of a system of molecular patterns and relations around them. Such context dependency should not be discouraging, as the dynamical aspect of biochemical phenomena should allow for no strict requirements on precise material barriers. If the material identification of the gene is problematic, we can still retain that transcription happens in particular contexts and not all parts of the DNA are transcribed. These complexities are the starting point from which I will argue that genes are novel and robust, and so emergent.

### The emergence of the gene

Following the definition of the postgenomic gene, a full understanding of it should consider its material composition in DNA sequences together with its functional significance. The utility and relevance of this definition, together with the three macro-patterns identified before, will be used to argue that **Ft** is novel and robust. As a result, I will conclude that the gene is emergent.

Here, I will first briefly present the precise context I will use to support the thesis. Then, I will consider why the functional property defining the genes can be considered novel and robust. A consequence will be that genes emerge during gene expression, that is in the moment in which transcription is happening, and they are constituted by *some* nucleic acids. However, this proposal remains weak and coherent with non-eliminativist ontological reductionism. When I write of genes as emergent entities, I do not mean they are concrete separated individual objects, but simply that they exist as something qualitatively distinct from the mere DNA bases. The components of the gene are identified with specific tokens of chemical molecules involved at the time considered, but they are not any molecules but the ones with the property **Ft.**^26^ This view implies a temporal and contextual connotation of the gene, as its weak emergence is supported by the one of **Ft** realised specifically during transcription. Moreover, this is coherent with genetic practice as often the DNA is manipulated to manipulate the genes, assuming a token identity between the lower-level and the emergent one, despite a difference in type.

#### Genes and protein synthesis

Even if genes can also encode RNA molecules with regulatory and functional roles within the cell, their scientific importance is particularly significant in consideration of protein synthesis. Thus, I exemplify my argument focusing specifically on genes that encode the primary structure of a polypeptide. Protein synthesis is the entire process that produces proteins inside the cell. It can be divided broadly into two main phases: transcription and translation. Transcription is the first phase in which a section of DNA “becomes” the gene: a sort of template molecule, or “image”, for the messenger RNA (mRNA). The transcription of a gene into a mRNA sequence (which can be then further elaborated into a mature mRNA) is carried out by RNA polymerases. The action of this enzyme is possible firstly thanks to the detection of the promoter region and the action of enhancer or silencer regions (Griffiths & Stotz, 2013). Then, it is dependent also on molecular conditions of the relevant section of DNA, such as chromatin remodelling and the action of other proteins, and general cellular interactions. Translation is the second phase in which the mRNA is translated by the ribosomes that use the sequence to order the sequence of amino acids for a polypeptide chain. For the gene’s analysis, let us focus on transcription. As already underlined, the gene has a specific existence within the right cellular environment, and it is intertwined with its transcription. The complex context in which transcription happens makes the core property of the gene **Ft** relational, as it needs a set of interactions for it to be present. This is pivotal to understand genes’ emergence. The gene is existent thanks to the action of RNA polymerases, together with promoter regions and other regulatory regions of DNA, the right cellular environment and the unfolding of DNA. Let us now consider more in detail the reasons why the genes satisfy novelty and robustness and can be considered properly emergent during protein synthesis.^27^

#### Novelty

As aforementioned, novelty is i) what makes it possible to identify the emergent property in a distinctive way from the properties held by the lower-level entities; and ii) the consideration of such a property improves explanations, leading to new ones. The novelty of the definitional property of the gene **Ft** is found in both these aspects.

First, that **Ft** is not a property of precise stretches of DNA with definite barriers^28^ should be clear from the *multiple realisablity* of **Ft** and the *multiple constitution* of the gene mentioned in **4**. Genes result to have a discontinuous structure that can make one gene to be completely contained inside another one’s intron, or one gene to overlap with another (Gerstain et al., 2007). Considering any specific transcribed product, this can be realised by different stretches of DNA and, conversely, different stretches of DNA can realise similar products. **Ft** is present when there are complex interactions between different parts of the genome, as gene regulatory networks, and depends on the action of RNA molecules and enzymes. This property is specifically of the gene and it is what distinguishes other regions or sequences of DNA from genes regions.

Second, the novelty of **Ft** is justified by the improvements the gene brings to our explanations in science. If the structural-material conception of genes, for which genes are defined only by their material DNA components, cannot account for the complexity of the transcription and the first stage of protein synthesis, the consideration of its functional property helps scientific practice. Interesting cases are represented by monogenetic diseases or alternative splicing. It is thanks to the conceptualisation of the gene as a functional unit that “mirrors” a molecular product (even if more or less), rather than a “mere” straightforward DNA stretch, that one can explain and discover these complex phenomena or the interactions among different components necessary to encode a polypeptide. Even more, it is the conceptualisation of the gene as a functional unit that allows to see its importance at the cellular and organismal level.

As mentioned earlier, one of the main explanatory roles played by gene is the one they have during protein synthesis. Genes are the basic building blocks to explain why some proteins are encoded rather than others and allow for the conceptualisation of the transcription of some parts of DNA rather than others (even if they are not the only main agents^29^). This role is important in different domains, from molecular biology to medicine. For instance, genes have an important explanatory role in the case of the monogenetic disease cystic fibrosis. In this disease, the mutation of a single gene *CFTR* on chromosome 7 in humans can cause a disruption in the production of the Cftr protein, which regulates the flow of salt and fluids in and out of the cells and regulates the levels of mucus within the body. The consideration of this single gene, as a combination of the sequence and specific **Ft** allows for a better explanation. The mutation in the gene *CFTR* alters the result of the final molecular product, as it is a different “image”, and explains the possible disease. In “normal” cases, on the contrary, it is the absence of such mutation in the gene *CFTR* that allows to explain the normal levels of Cftr proteins. Scientists and doctors speak of mutations within the gene (and not sequence-mutation) and of *mono-genetic* disease because it is the consideration of such a single gene (with the mutation) that explains the presence of the disease.

The novelty of the gene can also be noticed considering a lower-scale genetic phenomenon, such as alternative splicing. In this phenomenon, the patterns of introns and exons are rearranged such that the same DNA stretch can be transcribed into different mRNAs that encode different proteins. Nevertheless, this case is often considered one of a single gene if the proteins encoded are functionally similar enough and so they can both count as “mirroring” the same gene (Fogle, 2010; Gerstain et al., 2007; Griffiths & Stotz, 2013). Again, the functional aspect plays an important role. If one would consider only the material stretch of DNA, alternative splicing would be a bizarre phenomenon in which the gene is cut and modified to produce different products.^30^ With a partially functional view of the gene, however, one can theoretically elaborate alternative splicing as a case in which the same gene can be transcribed in different RNAs, realising different (but similar) functional outcomes^31^(see Fogle, 2010). Moreover, **Ft** explains why certain polypeptides are obtained from alternative splicing, instead of others, and can even help the prediction of these results.

**Fig. 1:**
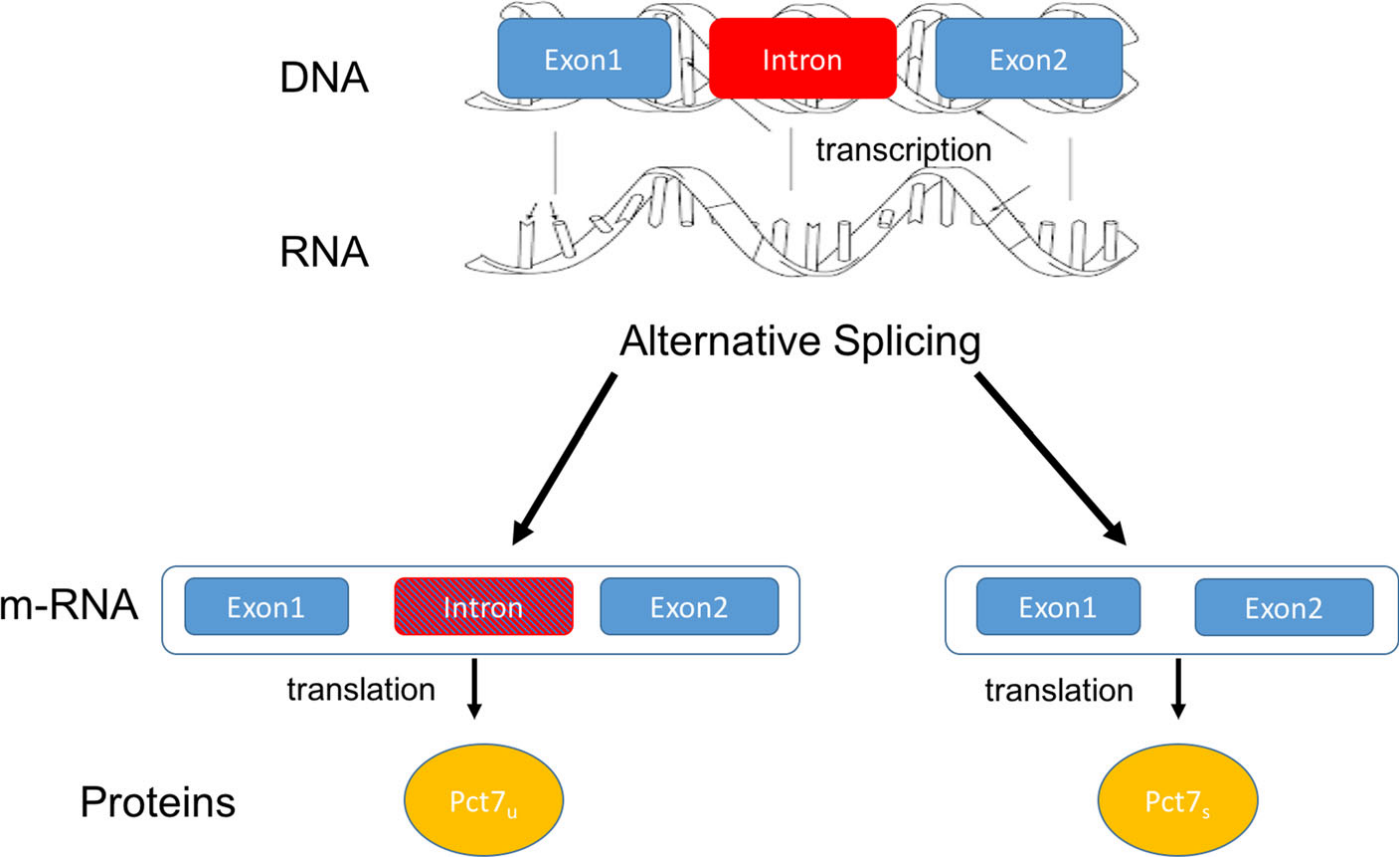
Alternative splicing in *PTC7* in *Saccharomyces cerevisiae*. This is a simplified representation of alternative splicing at the *PTC7* gene and the encoding of the proteins *Ptc7*_u_, with the intron, and Ptc7_s_, without the intron. It helps visualising the difficult identification of the gene with a precise stretch of the DNA and its multiple determinability. For the full information about alternative splicing of *PTC7*: Juneau et al. (2009)

Alternative splicing is a highly frequent phenomenon and one elucidating example is given by the *PTC7* gene in the genome of *Saccharomyces cerevisiae.*^32^ The *PTC7* is a gene that, after the transcription in RNA, can be alternatively spliced to generate two different mRNAs that can be translated into distinct, but functionally similar, proteins. In particular, the relevant gene is transcribed into two mRNAs, codifying different proteins, Ptc7_s_ and Ptc7_u_ [Fig. 1]. In the case of *PTC7*, “one isoform of *PTC7* is created by the removal of the intron, while the other isoform results from intron retention” (Juneau et al., 2009, 186). This case of alternative splicing supports the novelty of **Ft**. It is thanks to the functional properties of making two different, but similar proteins (**FPtc7**_**u**_, **FPtc7**_**s**_) that it is possible to conceptualise alternative splicing as the presence of two different products of the same gene. Moreover, alternative splicing illustrates the multiple determinability of the DNA that makes different proteins.

On the grounds of these phenomena, I can conclude that the gene with its property **Ft** is novel as it displays qualitative difference and the consideration of it improves scientific explanations.

#### Robustness

Further, genes can be considered a relatively robust phenomenon. Robustness is the core ontological feature of emergence, as a guide to ontological commitment and as a further support to novelty. As aforementioned, a phenomenon is robust when it displays stability within a certain range of perturbations and its properties are realised even if the system undergoes some modifications (Butterfield, 2011; Franklin & Knox, 2018).

In the genetic context, perturbation can be read in terms of mutations or changes that can affect the realisation of **Ft** and the transcribability of a given region of DNA. This type of robustness can be indicated as a form of *functional robustness*^33^ that is “the robustness of some function or effect produced by a system over variation in or perturbations to the components and properties of that system” (Boone, 2018, 81).^34^ Briefly, this means that the functional property is realised despite a range of modifications at the underlying lower level. Robustness is so related to multiple realisation, through which the property is realised by multiple lower-level features, and to multiple constitution, for which the phenomenon defined by the functional property can be constituted by a variety of bases within different circumstances (Boone, 2018; Gillett, 2013; Kistler, 2018). In order to understand the robustness of the gene, let us recall the definition offered by Gerstein et al.: “*A gene is a union of genomic sequences encoding a coherent set of potentially overlapping functional products*” (Gerstain et al., 2007). As evident from the relations mentioned in **4.1**, **Ft** of a given union of genomic sequences (the gene) is robust within limits of perturbations of the DNA stretch. This is because there are many complex mechanisms, such as reparatory or alternative ones, that maintain the transcription of a given set of potentially overlapping functional products stable. Consequently, the gene is said to be robust because it can be constituted by different stretches of the DNA and obtains despite some ranges of modifications in its components. Robustness is evident in a variety of genetic phenomena and I will consider some of them in what follows.^35^

**Fig. 2:**
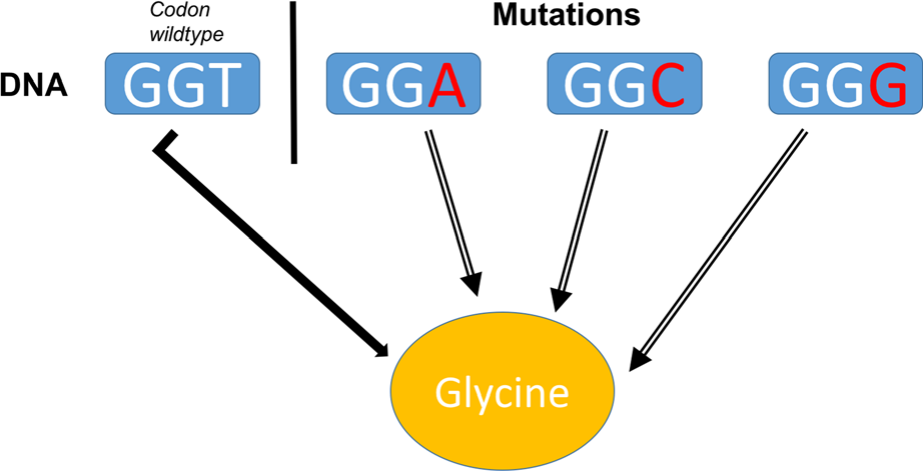
Silent mutations in codons for the amino acid glycine. This is a simplified representation of the modification of a base in the wild type, GGT, into three silent mutations. The black arrow stands for codification in the wildtype, while the empty black arrows stand for codification in the cases of mutation. The amino acid is made in all four cases. The gene that encodes a protein containing glycine and the function remain robust, because the function is realised even in presence of mutations. The figure helps visualising the *multiple realisablity* of the functional property of the gene, in this case the property “encoding a protein with glycine”

A first evidence comes from “synonymous mutations”, changes in the DNA sequence that codes for a specific amino acid without affecting the final product, the encoded amino acid. This is possible thanks to the redundancy of the genetic code, an adaptive feature for which multiple codons can code the same amino acid. Broader synonymous mutations can also code for the same polypeptide, supporting the robustness of the functional property **Ft**. An interesting example in this regard is glycine, a proteinogenic amino acid (an amino acid that is integrated into a protein during translation). Glycine is codified by GGT^36^ and any change in the third position of the codon, either with A, C or G (resulting in GGA, GGC, and GGG) will result in the same amino acid in the right position, even when coding a more complex protein sequence (Waters et al., 2016, 3) [Fig. 2]. This phenomenon is called single-nucleotide polymorphism, because the mutation involves only a single change in the codon. This synonymous mutation is simple, highly frequent and shows a first level of robustness across perturbations.

Moreover, robustness is found in broader phenomena where the context and mechanisms around the gene allows for the property **Ft** to be present despite changes in the DNA. Cells have evolved an “orchestrated interplay of various DNA repair mechanisms to prevent the life-threatening disruption of replication and transcription by DNA damage” (Walmacq et al., 2012, 1). This type of robustness represents a necessary feature for the cell to perform protein synthesis and other activities: if the cell were to be disrupted by any sort of underlying perturbation, it would probably stop to live very soon. As illustrated by Griffiths and Stotz (2013), the genome is highly reactive to a system of different mechanisms that allows for the transcription of the gene and maintains such transcription stable despite underlying perturbations. In so far as the transcribed products are potentially overlapping and functionally similar, the considered union of genome sequences can be deemed as the same gene (as Gerstain et al., 2007, Fogle, 2010). An instance where robustness is evident is in mechanisms of translesion transcription by RNA polymerase II in *S. Cerevisiae*. In these cases, the translesion RNA polymerases II is able to transcribe the gene with or without repair of the damages created by exposure to UV lights (Walmacq et al., 2012).^37^ This is relevant because it allows for the property **Ft** characterising the gene to be nevertheless realised, despite the underlying modifications. Furthermore, given the relational nature of such a property it shouldn’t be surprising that this realisation happens thanks to the interaction with an external actor, the RNA polymerases.

To sum up, all these cases of robustness are accounted for by the multiple realisability of **Ft**, the multiple constitution of the gene and the multiple determinability of the DNA. Such multiple realisation and multiple constitution concerning genetic phenomena are possible thanks to all the interactions that are pivotal to the defining property of the gene **Ft**. The possibility of transcription becomes real within the right environment and system of relations.

The robustness of the gene has interesting consequences for the tensions concerning the gene concept. First, robustness is a feature present thanks to a full set of interactions and mechanisms that surrounds the gene and allows its stability. This is line with the idea that the identity of the gene could be understood only within the right context, the cell and the overall environment. The defining property of the gene **Ft** is a relational property and this makes it dependent on different contexts. Such context dependence of the gene should not represent an obstacle to its existence, but it should be rather accepted as a crucial feature of its identity. Second, the gene’s existence has a temporal connotation. Using Griffiths and Stotz’s expression: “genes are ways in which cells utilise available template resources to create the biomolecules that are needed in a specific place at a specific time: genes are things an organism can do with its genome” (Griffiths & Stotz, 2013, 75; also in Griffiths & Stotz, 2006). The definitional property of the gene, being relational, can only be realised when there is the possibility for such relation to hold and when there is the need for it to hold. This implies that the gene itself exists within protein synthesis or, more general, within the process of transcription. Thus, it is dependent on a variety of different mechanisms that make the genome react in different ways, allowing for the emergence of the genes in specific moments.

Taking stock, a gene emerges from a precise stretch of DNA when it is transcribed, and this happens when complex mechanisms are acting in the surrounding context. It is this possibility that permits its novelty in scientific explanation and it is such a system of mechanisms that allows for its robustness.

## Conclusion: flexibility and context dependency

The account presented in this paper wants to accommodate the tensions concerning genes identity and existence. Specifically, these tensions are generated by the complexity of the genetic phenomena, as highly context-dependent, and the importance that genes have across different biological disciplines, from molecular to medical biology. Here, I have argued that they can be solved by elucidating the existential characteristics of genetic phenomena^38^: genes are existent weakly emergent entities. This result has been reached in two steps. First, I have clarified and defended a definition of postgenomic genes as “images of the molecular products”, re-elaborating the ones of Gerstain et al. (2007) and Griffiths and Stotz (2006, 2013). Then, I have argued that genes result to be emergent as they display novelty and robustness. A consequence of this is that a gene emerges during transcription. Furthermore, this emergence remains *weak* since it is compatible with a form of ontological reductionism. Thanks to the token identity between the gene and the DNA, we can maintain it as materially constituted of nothing more than chemical molecules, but the straightforward linear relation between DNA segments and genes is untenable. The gene has a proper type identity that is given within the cellular environment.

The emergence of genes is an important conclusion as it offers a new conceptualisation of the complex genetic phenomena, allowing us to accept their existence despite the unclear material constitution and the multiply-realised transcription factor. Postgenomic genes exist within the different circumstances in which transcription happens and the identification of the precise basis should be highly flexible (Fogle, 2010). Such a flexibility, crucial for the topic, cannot be accounted for if genes are considered merely materially, as mere ORFs. Nor we have to be satisfied with the simple nominal and operational definition of the genes. On the opposite, we might consider emergence as what allows the existence of the genes together with more flexibility in defining their borders and it permits the consideration of their core relational and context-dependent property. Genes can thus result to be both flexible and highly context-dependent, and nevertheless existent, as they depend on the right environment that allows their definitional property to be robust. Furthermore, this account provides an ontological and not merely theoretical reason for which gene-talk in life sciences is justified as well as the important role that genes play in contemporary biological disciplines. If one of the aims of science is to unmask which kinds of things exist in the world, then the present account offers more support to the fact that science has reached this goal. Second, the importance of considering the context-dependency of genes might be relevant for those scientists that are trying to conceptualise and model genetic phenomena.

At last, this case can be of interest to philosophers working on emergence in the sciences. It presents the gene-case in support of a recently proposed account of ontological emergence that has been firstly applied to physical phenomena but had the aim of being more general. Particularly, it fits biological cases thanks to the relevance of robustness and its attention to the importance that specific scientific concepts have in explanations.

## Footnotes

1 I thank reviewer 3 for the insightful comments on this introduction.

2 Reference for moderate naturalism is Tahko and Morganti (2017). According to this approach, ontological considerations should be done on the basis of a dialogue between metaphysics and the scientific postulation of entities.

3 Further analysis will be provided in **3**, **4.1.3**.

4 I thank reviewer 1 and Dr. Margarida Hermida for pointing out that genes can be composed of both DNA and RNA accordingly to the phenomenon or the account under consideration (as in Scherrer & Jost, 2007). Specifically, Scherrer and Jost (2007) suggest that genes as images of the final molecule emerge in eukaryotes in the final mRNA. Or, if we accept that also viruses have genes, they might be composed of RNA. For simplicity’s sake, throughout the paper I will consider genes as composed of DNA, being aware that they could also be composed of other nucleic acids such as RNAs.

5 See Fogle (1990) for the different models of what constitutes the material component of the genes.

6 Context does not mean different disciplines or different scientific practices, but different environmental contexts such as the nucleus, the cell and the overall organism with its environment. As in Griffiths and Stotz (2013), the regulatory molecules and mechanisms are intra-cellular, inter-cellular and extra-cellular environmental systems.

7 It is common to advocate for a pluralist view of genes for which there are a variety of valid gene concepts in various disciplines, as sustained by Hall (2001), or Fogle (2010) and as reported by Griffiths and Stotz (2006, 2013). For instance we can distinguish “instrumental genes”, as “factors in a model of the transmission of a heritable phenotype” from the aforementioned “nominal” and “postgenomic genes” (as in Griffiths & Stotz, 2006).

8 Another source of reference for the discussion on the definition of genes in textbooks is Gericke et al. (2014), as suggested by reviewer 1.

9 Many have tried to account for the precise material basis of genes. Fogle (1990) proposed 4 different models to accommodate the concept of gene: Model A includes the transcribed region of the DNA and all neighbouring sequences which influence the process; Model B considers only the transcribed region; Model C includes only the set of exons derived from a pre-mRNA; Model D is limited to the coding exons of a primary transcript, excluding non-coding leader and trailer sequences. Here, I leave open the question about the exact basis from which the genes emerge.

10 A gene is different from other merely functional kinds, such as chairs or bicycles, which are what they are only because they perform a certain function.

11 I consider the general kind *gene*, even if in nature there are specified genes with specific functional properties.

12 I am aware of the difficulties of defining emergence in an unambiguous way, as reported in Wilson (2015, 3). This section does not have the ambition of elucidating the general discussion on emergence and its metaphysical significance.

13 This account is also compatible with forms of epistemological or methodological reductionism, but this will not be considered here.

14 Ontological reductionism is considered compatible with realism about a phenomenon as in Brock and Mares (2014).

15 Provided that the lower-level features are physical, this position can also be referred to as non-reductive physicalism. Moreover, the novelty of a phenomenon and its emergence does not lead to “any kind of failure of theoretical reduction” (Franklin & Knox, 2018, 74).

16 The term entity is left willingly unspecified, as it can be whatever bears a property according to the ontology considered. An entity can be a concrete particular object, an abstract one, a process or an event.

17 This precision of terminology is justified by its coherence to scientific practice as in Gillett (2013).

18 It is legitimate to ask about the relation between these features and the phenomenon’s being emergent. Here the two features, novelty and robustness, should be considered the *ratio cognoscendi*, i.e. how to know that something is emergent. But from the ontological point of view, it is the fact that the phenomenon is emergent that represents the *ratio essendi* of the features, novelty and robustness. We can know that something is emergent when it displays novelty and robustness. But something displays novelty and robustness because it is emergent (see also Eronen, 2015, 3966). To summarise, emergence represents the feature thanks to which we can both postulate the existence of something and its knowledgeability. So, when studying an emergent phenomenon, we can know that it is such because it displays novelty and robustness: we have epistemic access to its emergence thanks to its being novel and robust. But, from an ontological point of view, the phenomenon can display novelty and robustness because it is emergent, and so existent, in the first place. I thank reviewer 3 for pushing me on this point.

19 I thank reviewer 1 for pointing out that improving explanations and providing new ones are two different epistemic moves. However, if we are considering explanations about the *same* phenomenon, then the difference between the two might be on a continuum. Here novelty is interpreted as what leads towards novel explanations and these can be seen as an improvement or “empowerment” of the explanations already present (as in Franklin & Knox, 2018). These improvements might also lead to new explanations about the same phenomenon. Although this raises some interesting issues, this topic would require a separate paper and there is no space to develop it in more detail.

20 As pointed out by reviewer 3, novelty can be provided also by idealisations that play an important explanatory role in science. However, idealised phenomena are not what usually count as possibly emergent. This is one of the reasons why the feature of novelty is not sufficient on its own to provide emergence and it has to be combined with robustness. Much more could be said on the role of idealisations in science and their ontological impact, but this topic cannot be explored here due to the focus of this paper.

21 In Franklin and Knox: “To show that a phenomenon is robust is to show that its description and dynamics are stable with respect to perturbations in the underlying physics; in order to be emergent, a phenomenon must not be too fragile or too fleeting.” (Franklin & Knox, 2018, 73).

22 For multiple constitution see Gillett (2013) and Kistler (2018). In Kistler, a phenomenon is multi-constituted when there are two or more microscopic structures that obtain the phenomenon (Kistler, 2018, 18).

23 This property is identified *ahistorically*, that is genes are (mostly) individuated by what they do now, rather than their evolutionary history (the distinction from ahistorical and historical individuations is from Gillett, Forthcoming). As indicated by reviewer 3, the material identity of the gene is also ontologically relevant and represents a condition of being. However, it is not sufficient on its own to identify the genes, given that genes are not only stretches of nucleic acids, but specific ones that are “images” of a given molecule. Both aspects of the gene definition (the material and the functional one) are ontological, but given that the functional one is what allows us to distinguish what is a gene from what is not, I have focused the discussion on emergence on the functional component.

24 For the importance of functional similarity of products in genes classification see Fogle (2010).

25 On the lack of straight linear correspondence between bio-molecular entities and functions see also Kistler: “There is a second and complementary reason for which there is no 1:1 relation between biomolecules and functions. The first was that a given type of molecule can have several functions. The second reason is that several molecules can share a function.” (Kistler, 2018, 16).

26 The author considers the identification of the exact bases from which genes emerge an empirical enterprise determined by scientific practice.

27 As suggested by reviewer 3, the process of transcription can be identified as the ground of becoming or *ratio fiendi* of the gene that emerges during this specific process and in a dynamic context.

28 Claiming that **Ft** is not a property of a precise stretch of DNA *does not* imply that it can never be considered, for scientific practice or in simple cases such as in prokaryotes, a property of a precise stretch of DNA. According to weak emergence, the property and the emergent phenomenon need a synchronic basis, in this case the DNA molecule, from which they are realised and in simple cases the identification of the gene with a well delimited stretch of DNA can work. Nevertheless, a strict identity relation rules out many interesting genetic phenomena and is incompatible with the assumed functional definition of the gene (see Fogle, 2010; Griffiths & Stotz, 2013).

29 See Griffiths and Stotz (2013) for a more detailed analysis.

30 El-Hani writes “alternative RNA splicing requires that the conceptualization of a gene [i.e. material gene] moves far beyond the simple scheme captured in formulas such as one gene [i.e. material gene]-one protein or polypeptide” (El-Hani, 2007, 4).

31 As pointed out by reviewer 1, the interpretations of alternative splicing are complicated due to the issue of “similarity” between the functional outcomes. If the functional outcomes are similar enough, then there is one gene mirroring two different products, while if the similarity is too weak, then it can be a case of two genes. However, given that similarity is a vague relationship, drawing the line should be done case by case and should be based on scientific practice. What remains relevant for the current discussion is the importance of Ft for the identity of the genes.

32 Another interesting case is the *DSCAM* in *Drosophila* (Kashyap & Tripathi, 2008). *DSCAM* contains 116 exons of which 17 are maintained in the final mRNA. Kashyap and Tripathi (2008, 3) write that “theoretically this system is able to produce 38,016 different proteins. And, in fact, over 18,000 different ones have been found in *Drosophila*”, supporting the multiple determinability of the stretch and the realisation of different functional products.

33 This is a type of *structural robustness*, as a change in the attributes relevant to the structure of the system under consideration, and in the biological cases, it includes the environment as well (Weisberg, 2013, 161). It differs from *parameter* robustness, i.e. changes to the parameters of the model’s description, and *representational* robustness, i.e. changes to the representation of the attributes in a model (Weisberg, 2013, 160–163).

34 This elaborates on Mitchell (2008, 698), where robustness is defined as “the ability of a biological system to maintain normal functioning in the face of internal or external perturbations”.

35 Note that this section is not arguing that the gene is more robust than the DNA, but that the gene displays some robustness.

36 This is the most common wildtype for glycine, even if there are variations across different species. For instance, some Bacteria will use GGA more often.

37 UV exposure can cause cyclobutane pyrimidine dimers (CPDs) in the DNA strand that stall transcription elongation by RNA polymerase II (Pol II). For full reference on translesion RNA polymerases II see Walmacq et al. (2012).

38 I thank reviewer 3 for these comments.
